# Book Notices for March

**Published:** 1869-03

**Authors:** 


### BOOK NOTICES FOR MARCH.

CONTENTS OF HALL'S JOURNAL OF HEALTH FOR JANUARY, 1869. Tickling in the Throat; Stomach Cough; Lung Cough; Throat Cough; Cough Curative: its Supression Mischievous. House Servants; Kitchen and Parlor Daughters; Premature Breaking Down; A Good Cook; Throat Disease; Chapped Hands; Crooked Fingers; Nuts and Cheese; Christian Associations; Dangerous Medication; Sacrificed Lives.; "Liebig's Extract of Meat."

FEBRUARY. Essence of Disease; Shocks; Selfishness; Washing the Face and Hands; Tight Boots; How to Make a New Shoe Fit Easy. Breathing; Cold Feet; Slippers; Health and Thrift; Unhealthful Houses; Sleeping Habits; Fire on the Hearth; Kerosene Fatalities.

MARCH. "Protestantism a Failure;" A Common Cold; Wakefulness; Common Sickness; Where to Live Cheaply; Schools and Living in Heidelberg, Manheim, Dresden, Bonn; Cost of Travel up The Rhine; The Unfortunate Poor; Buggy Sugar; Soap-suds; PoisonousMoulds; Bed-bugs; Umbrellas Dangerous; The Early Dead, etc.

DR. J. S. WILSON, author of "Woman's Home Book of Health," and formerly a contributor to the health department of Godey's Lady's Book, of Philadelphia, is now contributing a series of short articles on health in the Houston (Texas) Telegraph, which are expected to be useful and interesting to all, especially to the ladies, bless them.

THE GREEN MOUNTAINS. According to some "vital statistics" of Vermont, it is about the healthiest and wickedest community on the globe; the healthiest, because in one year the deaths were only a fraction over twelve persons out of every thousand; the death-rate is three times as many in New York city; the most wicked, because there was one divorce in every twenty of the marriages; what is New England coming to? In Vermont the people are getting unmarried at a fearful rate; in Massachusetts they are about hiring persons to get married, and in other parts of Yankeedom they are "putting away" babies, to such an extent, that an Episcopalian Bishop in New York city has fulminated his malediction, in a pastoral letter against a practice alleged to be so alarmingly common. Human laws will never be able to correct these demoralizations; the simple education of the masses will not correct them; nothing can avail but the early introduction of the principles of the Christian religion in the minds, hearts and consciences of the young, and continued through life, and through the instrumentalities of the house of God on the Sabbath day.

